# New structure enlivens interest in P2X receptors

**DOI:** 10.1016/j.tips.2010.02.004

**Published:** 2010-05

**Authors:** Liam E. Browne, Lin-Hua Jiang, R. Alan North

**Affiliations:** 1 Faculty of Medical and Human Sciences, University of Manchester, UK; 2 Faculty of Biological Sciences, University of Leeds, UK

## Abstract

P2X receptors are ATP-gated membrane ion channels with multifarious roles, including afferent sensation, autocrine feedback loops, and inflammation. Their molecular operation has been less well elucidated compared with other ligand-gated channels (nicotinic acetylcholine receptors, ionotropic glutamate receptors). This will change with the recent publication of the crystal structure of a closed P2X receptor. Here we re-interpret results from 15 years of experiments using site-directed mutagenesis with a model based on the new structure. Previous predictions of receptor stoichiometry, the extracellular ATP binding site, inter-subunit contacts, and many details of the permeation pathway fall into place in three dimensions. We can therefore quickly understand how the channel operates at the molecular level. This is important not only for ion- channel *aficionados*, but also those engaged in developing effective antagonists at P2X receptors for potential therapeutic use.

## Introduction

Like many other transmitters, extracellular nucleotides act on two types of cell membrane receptor. These are ion channels (termed ‘P2X’) and G protein-coupled (termed ‘P2Y’) receptors [1]. P2X receptors first came to prominence as the ligand-gated ion channels involved in transmission from sympathetic nerves to smooth muscle effectors [2], just as nicotinic acetylcholine receptors were discovered by their role in somatic neuromuscular transmission [3], and glutamate receptors for their analogous function in the central nervous system [4]. It is now clear that for P2X receptors this fast signalling represents only one end of a broad functional spectrum. It is also apparent that in most tissues nucleotides act as paracrine transmitters that diffuse considerable distances and act over several seconds rather than a few milliseconds. Over the past decades, interpretation of the functional roles of P2X receptors has been handicapped by a lack of specific pharmacological tools. It has been further complicated by the discovery that some P2X receptors (notably P2X7) do not simply open a narrow conducting pathway to allow the passage of small ions, but also become progressively permeable to larger cations such as N-methyl-D-glucamine and propidium dyes [5].

P2X receptors are widely distributed in mammalian tissues. Seven subunits are encoded in mammalian genomes, and these form channels as homotrimers or, in a few circumstances, as heterotrimers (e.g. P2X2 and P2X3, P2X1 and P2X5) [6]. They fulfil roles that extend from thrombosis through gastrointestinal motility, afferent sensation (including chemoreception and taste), renal autoregulation and bone resorption, to inflammation [6–8] (Table 1). Such roles have now been firmly validated by studies in mice in which one or more of the subunits has been genetically eliminated. Accordingly, exploitation of these potential new therapeutic targets has focused on P2X1 receptors for disorders of the urinary tract; P2X3 and the heteromeric P2X2/3 receptors in pain; P2X4 receptors in neuropathic pain; and P2X7 receptors for blocking the release of inflammatory cytokines [9] (Table 1).

**Figure 1:**
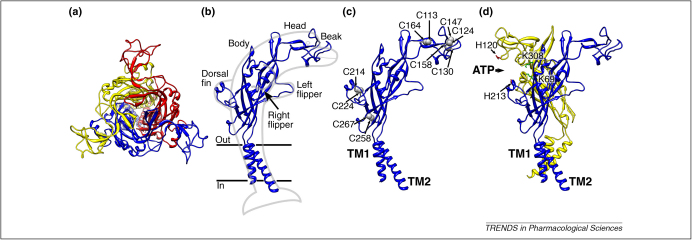
Three subunits form one P2X receptor. **(a)** The trimeric rat P2X2 model, viewed along the axis of threefold symmetry from the extracellular side, with each subunit depicted in a different colour. **(b)** A single P2X2 subunit, viewed parallel to the membrane plane with the outline dolphin suggested by Kawate *et al.*[10]. **(c)** Depiction of the five disulfide bonds within a single P2X2 subunit. Sulfur atoms are shown in grey. **(d)** Two subunits of the P2X2 receptor presented so as to emphasise the ATP binding pocket which they jointly form. This and subsequent figures show homology models of the rat P2X2 receptor generated with Modeller 9v7 [11] using the zebrafish P2X4.1 crystal structure (PDB accession 3I5D) as a template. MolProbity [12] was used to assess the five lowest-energy models: the selected model showed 98.9% of residues in the allowed regions of the Ramachandran plot. Images were produced using UCSF Chimera 1.4 [13].

cDNA cloning of P2X receptors in the mid-1990 s led to the deduction of a receptor structure that was very different from the ligand-gated ion channels then known (i.e. nicotinic receptors [including receptors for γ-aminobutyric acid, glycine and 5-hydroxytryptamine], and glutamate-gated channels). Each subunit had two membrane-spanning domains (TM1 and TM2), and the N-termini and C-termini were within the cell: the bulk of the receptor protein was extracellular (∼280 amino acids: the ‘ectodomain’; Figure 1a). In general terms, this resembles the topology of the epithelial sodium channel (ENaC) and its relatives in *Caenorhabditis elegans* (mechano-sensitive channels, the degenerins). However, except for the acid-sensing ion channels (ASIC), those channels are not usually considered to be ligand-gated [14].

The functional expression of these cDNAs has enabled a determined effort to deduce the molecular *modus operandi* of P2X receptors. There have been many studies using mutagenesis combined with functional expression, some showing considerable ingenuity. Taken together, these have allowed the initial designation of certain amino acids residues as being involved in specific functional aspects (e.g. ATP binding, ion permeation). The purpose of this review is to re-interpret such work in the context of the 3.5-Å structure of the zebrafish P2X4 receptor recently solved by Gouaux and colleagues [10]. This will assist understanding of the molecular detail of channel function, and provide a basis for the design and study of pharmacological agents. We have generated a model of the rat P2X2 receptor, which has been the principal subject of structure–function studies; residues identified in this article are numbered accordingly.

## How many subunits make a P2X receptor?

The careful work of Bean [15] on the initial slope of ATP dose–response curves prompted suggestions that P2X receptors open in response to the binding of three agonist molecules. Consistent with this, the availability of expressed proteins showed that the receptor was a trimer activated by three molecules of ATP (blue native polyacrylamide gel electrophoresis [16] and functional studies using Hill plots [17] or concatenated subunits [18]). Purified receptors observed by atomic force microscopy [19] and electron microscopy [20] also showed threefold symmetry.

The imagination of Oregon scientists [10] shows each of these three subunits as a dolphin rising from the surface of the ocean (cell membrane), with its tail submerged within the lipid bilayer (Figure 1b). The body regions of three such ‘dolphins’ mutually intertwine so as to surround a central vertical cavity (Figure 1a). The arching body of the dolphin allows the head/beak region to position more horizontally, lying across and making contact with the body of a neighbouring subunit. In the head and beak region are found three of the disulfide bridges previously predicted on the basis of mutagenesis (Cys^113^–Cys^164^, Cys^124^–Cys^147^, Cys^130^–Cys^158^) [21,22] (Figure 1c), whereas the Cys^214^–Cys^224^ disulfide is located within the dorsal fin. The Cys^258^–Cys^267^ disulfide bridge joins the ends of two β-strands low in the body region (Figure 1c). These disulfides bridges are not essential to P2X receptor function because most are not present in a *Dictyostelium* receptor (DdP2XA), which also forms an ATP-gated channel [23].

The three subunits have several regions of contact, some of which have been demonstrated through mutagenesis by the formation of ‘ectopic’ disulfide bonds. Thus, a disulfide bond is formed when cysteines are introduced at the positions normally occupied by His^120^ and His^213^: these histidines mediate binding and facilitation of P2X2 receptor currents by zinc [24] (Figure 1d). Hume and colleagues used concatenated subunits to show that these zinc-binding histidines are provided by different subunits [24] and put forward evidence that these as well as additional residues were probably close to the ATP-binding site [25] (Figure 1d).

**Figure 2:**
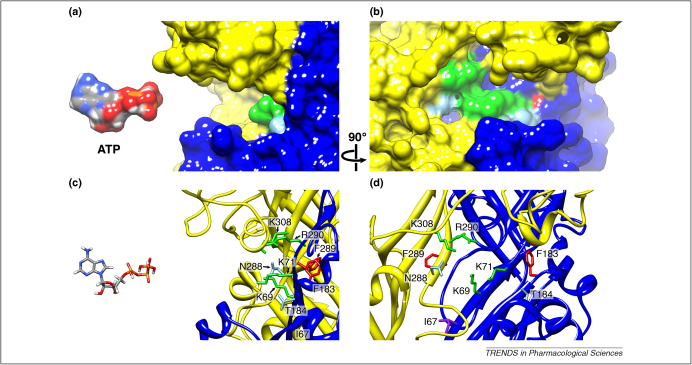
The ATP-binding site. **(a)** and **(b)** Space-fill models at 90° to each other viewed parallel to the membrane plane showing the binding pocket for ATP formed between adjacent subunits (shown in blue and yellow). Eight highly conserved residues positioned within the ATP binding pocket are indicated, with the respective ribbon diagrams shown in **(c)** and **(d)**. We speculate that the negatively charged phosphate groups may interact with the positively charged Lys^69^ and Lys^308^ residues, whereas Arg^290^ may interact with the adenine moiety. The ATP molecule (ligand from PDB accession 1B0U) is coloured by element. The amino acid side chains are colour-coded by property as follows: positively charged (green), aromatic (red) and hydrophobic (purple).

A second ectopic disulfide bond that has been reported is between cysteines introduced at the positions of Lys^69^ and Phe^289^ [26] (first shown for Lys^68^ and Phe^291^ in the rat P2X1 receptor [27]) (Figure 2). A third locus of inter-subunit contacts has also been demonstrated by formation of a disulfide bond between cysteines substituted for residues Val^48^ and Ile^328^ [28] located at the outer ends of TM1 and TM2, and channel opening occurs only when this disulfide bond is reduced [17,28]. An additional inter-subunit contact that has not been tested by mutagenesis is located between the short helix in the dorsal fin of one subunit and an exposed loop in the left flipper of another. The broad concordance with previous mutagenesis studies provides compelling evidence that the channel crystallised by Kawate *et al.* [10] corresponds to the closed state of a functional trimer that has been extensively studied in expression systems over the past 15 years.

## Where does ATP bind?

The structure solved by Kawate *et al.* [10] was in the absence of ATP, so we can not see directly where the agonist binds. P2X receptors do not exhibit any of the conserved sequence motifs that characterise nucleotide-binding sites in large numbers of intracellular proteins such as kinases. Conversely, a comparison of sequences that are distantly related through phylogeny (e.g. algae, slime mould amoebae, choanoflagellates [8]) but which are known to form functional channels shows that relatively few of the 280 residues of the extracellular domain are strongly conserved across all receptors (notably Lys^53^, Tyr^55^, Lys^69^, Lys^71^, Asp^82^, Gln^108^, Asn^182^, Phe^183^, Thr^184^, Leu^260^, Asp^261^, Asn^288^, Phe^289^, Arg^290^, Arg^304^, Leu^306^, Lys^308^, Tyr^310^, Gly^311^, Arg^313^, Gly^320^ and Phe^325^). Many of these residues have been suggested to be involved in ATP binding from functional studies.

The model structure places most of these conserved residues within or adjacent to a pocket formed between the body region of two subunits (Figure 2). Eight residues are most closely involved, four from each of two different subunits (Lys^69^, Lys^71^, Phe^183^ and Thr^184^ from one subunit, and Asn^288^, Phe^289^, Arg^290^ and Lys^308^ from another). Of these, two (Lys^69^ and Phe^289^) are sufficiently proximal to form a disulfide bridge when they are replaced by cysteine, and under oxidising conditions such a channel migrates as a trimer [26,27] (trimer formation is prevented if ATP is present, which was also consistent with these residues forming part of the binding pocket). A contribution to the ATP binding pocket by Lys^69^ and Lys^308^ from different subunits was first proposed by Wilkinson *et al.* [29], and this is now obvious in the model structure (Figure 2). Substitutions at these eight positions in P2X2 and the equivalent residues in other P2X receptors has previously been shown to inhibit strongly or prevent completely the action of ATP to open the channel (Table 2).

Jiang *et al.* [30] studied the effects of substitution at Ile^67^. They found that replacement with a positively charged residue (I67R, I67K, or I67C followed by a positively charged methanethiosulfonate (MTS)) reduced the maximal effect of ATP without changing the sensitivity (measured as the ATP concentration giving half the maximal current (EC_50_)). Introduction of a negative charge (I67E, I67D, or I67C followed by a negatively charged MTS) caused a parallel rightward shift in the ATP concentration–response curves. In pharmacological parlance, this latter inhibition was ‘surmountable’. It was deduced that Ile^67^ was very close to the ATP-binding site, and that the microscopic affinity for ATP was reduced by electrostatic repulsion. Consistent with this, the reaction of I67C with a negatively charged MTS was prevented if ATP was first applied. In the structural model, Ile^67^ is positioned at the lower lip of the ATP binding jaw, providing a good explanation for the conclusions of Jiang *et al.* [30] (Figure 2).

## Ectodomain binding of receptor antagonists

Models of mammalian P2X receptors based on the structure of the zebrafish receptor make clear what had been long suspected: the ectodomain presents a highly variable surface to the aqueous environment. Ectodomain sequences of the seven mammalian receptors range in pairwise identity from about 40% to 50%, which should provide reasonable scope for development of selective drugs. This goal has been achieved in several cases, but in only a few is there any clue as to the structural basis of selectivity. Suramin is one of the first P2X receptor antagonists. Suramin and its analogue NF449 appear to owe much of their high potency at the human P2X1 receptor (compared with that of the rat) to an interaction with Lys^138^ (Asp^136^ in rat P2X2) [31]. This lysine is positioned in a short helix that forms the lower part of the dolphin's beak, forming the upper lip of the ATP-binding jaw.

Most P2X receptors are blocked by pyridoxalphosphate-6-azophenyl-2’,4’-disulfonic acid (PPADS) but, when it was first cloned, the rat P2X4 receptor was found to be relatively insensitive [32]. A straightforward exchange of one lysine residue found in the PPADS-sensitive P2X2 receptor (Lys^246^) to the equivalent position in the P2X4 receptor (Glu^249^) transferred the PPADS sensitivity. Conversely, the substitution K246E in P2X2 converted the block by PPADS from essentially irreversible to quickly reversible. These results suggested that lysine at this position might form a Schiff base with the aldehyde moiety of PPADS, thereby preventing access of ATP to the receptor. The P2X2 receptor model places this lysine side chain extending in from the side of the mouth of the ATP binding jaw.

Michel *et al.* [33] showed that Phe^95^ in human P2X7 was required for high sensitivity to antagonists/allosteric modulators, including GW791343 (2-[(3,4-difluorophenyl)amino]-N-[2-methyl-5-(1-piperazinylmethyl)phenyl]-acetamide), SB203580 (4-[5-(4-fluorophenyl)-2-[4-(methylsulfonyl)phenyl]-1H-imidazol-4-yl]pyridine) and KN-62. The equivalent residue (Ile^94^ in zebrafish P2X4; Lys^88^ in rat P2X2) is found adjacent to a highly conserved proline deep inside the ATP binding cavity, where an attached antagonist might readily impede agonist binding. Determining the residues involved in the binding of the ever-increasing number of P2X receptor antagonists [9,33], and designing new selective antagonists *de novo* using *in silico* methods will be very important.

## How do ions permeate the channel?

Two main approaches, each combined with site-directed mutagenesis, have been used to identify the key regions involved in ion permeation. First, there are estimates of the relative calcium permeability of the channel (e.g. P_Ca_/P_Cs_ ratios or, more informatively, the fraction of the inward current carried by calcium (*Pf%*)). Second, the effects of applying MTSs on the ATP-evoked currents have been studied on channels in which a single amino acid has been replaced by cysteine.

### TM1 residues

**Figure 3:**
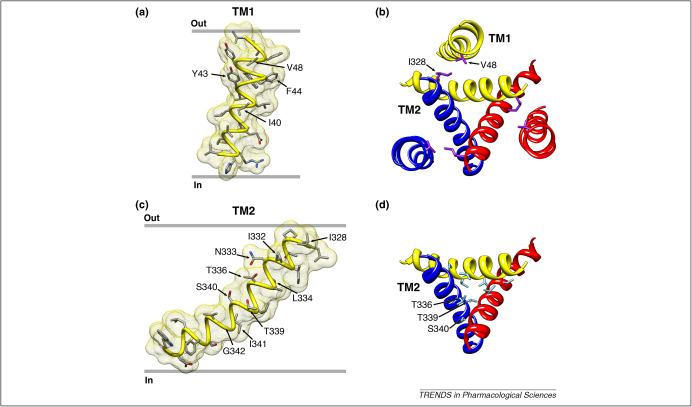
The transmembrane domains. **(a)** TM1 viewed parallel to the membrane with side chains coloured by element. **(b)** The close proximity of Val^48^ and Ile^328^ (purple) of TM1 and TM2 from different subunits, viewed perpendicular to the membrane plane from the extracellular side. **(c)** TM2 with side chains shown with standard element colours. **(d)** The narrowest part of the pore with polar gating residues Thr^336^, Thr^339^ and Ser^340^ indicated (light blue).

The first approach suggests that TM1 residues (Figure 3a) are not significantly involved in determining the relative calcium permeability of the P2X2 receptor [34], but there are two caveats to this approach. The first relates to Gln^52^ (of the rat P2X2 receptor). This residue is Glu in P2X1 and P2X4 receptors, and mutagenesis shows that in these receptors it is at least partially responsible for the high *Pf*% [35]. The conclusion that the negative charge of this Glu helps to concentrate calcium ions in the extracellular vestibule is well borne out by the structure. The second caveat pertains to Tyr^43^, which is conserved among all P2X receptors: removal of the Tyr at this position altered *Pf%* and P_Ca_/P_Cs_, but probably by an indirect mechanism rather than by contacting permeating ions [34]. In the model structure, this residue is well away from the permeation pathway. However, this central region of TM1 is at the closest point of interaction between the two crossing TM helices, and Phe^44^ and Ile^40^ from TM1 probably make hydrophobic interactions with Leu^334^ and Ile^341^ in TM2. The substitution F44C increases sensitivity to ATP [28], perhaps by facilitating inter-helix rearrangements during opening.

The second approach, the substituted cysteine accessibility method, also provided little consistent evidence that TM1 residues were exposed to the aqueous permeation pathway [28,36,37]. However, these results showed that Val^48^ (at the outer end of TM1) moves during channel opening so as to become more accessible to polar MTS compounds [28]. As mentioned above, a cysteine introduced at this position can disulfide bond with a cysteine at position Ile^328^ on an adjacent subunit (Figure 3b) and this disulfide bond inhibits channel opening [17,28].

### TM2 residues

The two approaches together have profoundly implicated TM2 (Figure 3c) in the aqueous permeation pathway. Thus, the relatively higher *Pf%* of P2X1 and P2X4 receptors appears to result (at least in part) from the acidic side chains provided by Asp in the outer vestibule [35] (this residue is Ser^326^ in the P2X2 receptor). Deeper within TM2, measurements of relative calcium permeability [38] and *Pf*% [39] clearly identify Thr^336^, Thr^339^ and Ser^340^ as providing side chains that interact with permeating calcium ions. The substituted cysteine accessibility method using MTS compounds and silver also place the same residues in the narrowest part of the permeation pathway [37,40,41]. The most recent studies show that the rates of modification of T336C and T339C (in rat P2X2) by 2-(trimethylammonium)ethyl-MTS (MTSET) are >1000- times faster when the channel is open than when the channel is closed [37], suggesting that the ‘gate’ is external to T336. Conversely, when cadmium is used as the probe, the gate lies between T336 and T339 [42]. Cadmium has an ionic radius similar to that of sodium, whereas MTSET is substantially larger. The model structure provides a plausible explanation for this because the side chains of Thr^336^ and Thr^339^ would be barely accessible in the closed channel (Figure 3d), even though they become accessible during opening. Given the differences in TM2 residues among species in this region (the corresponding residues are Ala^344^ and Ala^347^ in the zebrafish P2X4 receptor), further direct structural information will be required to resolve the side-chain orientations of TM2 residues in the open and closed states.

Three further approaches have been applied recently to the study of TM2: (i) appearance of spontaneous gating, (ii) changes in the unitary conductance, and (iii) alterations in open channel rectification resulting from the introduction of positive charges [43]. Almost any substitution introduced at Thr^336^ (including cysteine) results in P2X2 channels that are spontaneously active in the absence of applied ATP, suggesting that movements of this residue are critical to channel opening. At Thr^339^, only two such substitutions provided spontaneous activity (T339S and T339G) and a range of other side chains were tolerated. The introduction of a positively charged side chain, or cysteine followed by a positively charged MTS, at Thr^336^ or Thr^339^ also markedly changed the rectification normally exhibited by P2X receptor currents. Outward currents were strikingly increased, implying an altered interaction with permeating ions. Thr^336^ and Thr^339^ are found on the same face of the TM2 helix (Figure 3c), and form the narrowest part of the closed channel in the P2X2 model (Figure 3d).

Studies on Ser^340^ have been handicapped by the finding that the mutation S340C produces channels that do not respond to ATP [37,41] or respond with small currents [40]. However, Cao *et al.* [43] reported that channels with a positive charge introduced at this position also show markedly increased outward currents, and this could be mimicked in S340C by applying the positively charged MTSET. This observation indicates that the side chain at this position is also exposed in the permeation pathway, as initially suggested by Migita *et al.* [38] on the basis of their measurements of P_Ca_/P_Cs_. In the closed structure of the P2X receptor provided by Kawate *et al.* [10], the side chain of Ser^340^ (Leu^348^ in zebrafish P2X4 receptor) is pointing away from the central axis of symmetry (Figure 3c,d), which is presumed to form the open permeation pathway.

### Channel opening

The side chains of Thr^336^, Thr^339^ and Ser^340^ influence the movement of permeant ions (rectification, MTSET accessibility) [43]. This implies that the closed to open movement involves a counter-clockwise rotation by ≥50°. This would break the close apposition between Val^48^ and Ile^328^ of the neighbouring subunit mentioned above (Figure 3b). It would swing out of the way the hydrophobic side chain of Ile^332^ (equivalent to Leu^340^ in the zebrafish P2X4 receptor [10]) that appears to close the channel from the extracellular aspect (Figure 3c). This suggestion is compatible with the prediction of Silberberg *et al.* [44] that a rotation and splaying of the helices during opening could best explain the potentiating action of ivermectin at P2X4 receptors. The observation that the side chains of Thr^336^, Thr^339^ and Ser^340^ from each of the three subunits are exposed to permeating ions [43] suggests that TM2 helices not only rotate as the channel opens, but also become more steep from the relatively oblique angle (∼45°) at which they cross the membrane in the closed state [10]. Channel opening must also be associated with substantial movements of the inner part of the TM2 helices, which are splayed out in the closed state model (Figure 3), because MTSEA [40,41] and cadmium [42] block currents at rat P2X2 receptors that incorporate D349C. The irreversible coordination of cadmium by that receptor [42] clearly implies helical steepening, or perhaps bending at Gly^344^ [45], during opening. Structures of open and desensitized states are required.

The permeation pathway of P2X receptors undergoes a progressive dilation when the agonist application is maintained. This is most directly observed as a progressive increase in permeability to N-methyl-D-glucamine (NMDG) over several seconds [5,46–49]. NMDG is a monovalent cation that is too large (molecular weight, 196) to permeate the calcium/sodium/potassium-selective channel that opens when ATP is first applied. The slowly developing current (I_2_: seconds) can also be distinguished from the rapid current (I_1_: milliseconds) on kinetic grounds [46]. Khakh *et al.* [46] have shown that, in the case of the P2X4 receptor, point substitutions at position Gly^347^ (Gly^342^ in rat P2X2) alter the relative amplitude of I_1_ and I_2_; replacement with Tyr eliminates I_2_ but not I_1_, whereas substitution with Lys or Arg at this position selectively reduces the I_1_ component. They suggested that moving interfaces between the subunits may allow the progression from the I_1_ to the I_2_ state [50].

It is becoming increasingly clear from studying other ion channels (e.g. mechano-sensitive channels) that very large increases in size of the permeation pathway can result from tilting and/or rotation of helices [51,52]. The present structural model provides a firm basis on which to test the hypothesis that pore dilation results from progressive rotation and separation of the helix so as to open a larger permeation pathway. The first ‘shutter-stop’ of the three-helix iris might be a narrow channel selective to small cations; a second ‘shutter-stop’ might provide a channel that is also permeable to larger cations.

## Connecting binding to gating

**Figure 4:**
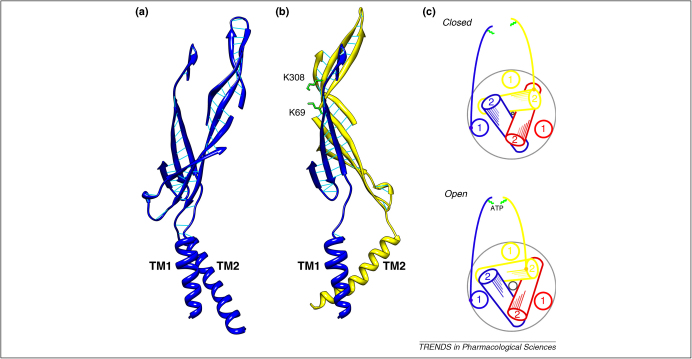
The connecting rods and a possible opening mechanism. **(a)** A single subunit viewed parallel to the membrane plane, with two connecting rods (the linkers and β-sheets) each rising from the TM1 or TM2 helices. **(b)** Two connecting rods from two different subunits, which contribute almost all of the eight highly conserved regions within the ATP binding pocket (including the critically implicated Lys^69^ and Lys^308^ residues). In (**a**) and (**b**) the backbone hydrogen bonds are shown (light blue) to emphasise the rigidity within each connecting rod compared with the limited contacts between adjacent connecting rods. Loops and helices that do not form the connecting rods have been omitted for clarity. **(c)** This highly schematic cartoon shows a possible mechanism for connecting ATP binding to channel gating. ATP binding (lower) repositions Lys^308^ relative to Lys^69^, and the connecting rod propagates a rotating and separating movement to TM2 relative to TM1.

The predicted ATP-binding site is 40 Å from the outer surface of the membrane bilayer. The two lysine residues most critically implicated in binding to ATP (Lys^69^ and Lys^308^, see above) are each about 20 amino acids removed from the outer end of TM1 (at Val^51^) and the outer end of TM2 (at Leu^327^). In each case, the structure linking the ATP-binding jaw to the outer end of the TM helix is a two- or three-stranded β-sheet, with a short linker at the membrane proximal portion; these are termed ‘connecting rods’ (Figure 4).

Within a single subunit, each connecting rod is relatively rigid due to backbone hydrogen bonding between β-strands (Figures 4a and b). There is also probably a hydrogen bond between the side chains of Tyr^55^ and Asp^261^ (two of the most highly conserved residues among the entire P2X receptor family). Tyr^55^ is at the linker extracellular of TM1, whereas Asp^261^ is located on a loop at the membrane proximal end of the TM2 connecting rod; mutation of either of these residues leads to complete loss of receptor function [28,40]. The strands also make contact in this region by a disulfide bond between Cys^258^ and Cys^267^ (Figure 1c). The model indicates minimal interaction between each connecting rod (Figure 4b), suggesting that they are free to move as they transmit the opening force from binding site to transmembrane helices, rotating and separating them as the pore opens (Figure 4c).

## Lessons for a broader channel family

Fifteen years ago, degenerins came to our attention because a mutant *Caenorhabditis elegans* was insensitive to gentle touch. This proved to result from a point mutation (Ala^673^ in Mec-10; Ala^442^ in Mec-4) that led to spontaneous activity of a mechano-sensitive channel, and consequent death of the few neurons that express the channel [53,54]. Epithelial sodium channels were discovered in the search for the molecular basis of high blood pressure (particularly Liddle's syndrome) [55]. Interest in the molecular identity of P2X receptors was driven largely by studies of chemical transmission in the autonomic nervous system [56–58].

These disparate physiological approaches have been brought together by the recent structural work from Gouaux and colleagues [10], which clearly shows that P2X receptors are related to the degenerin/ENaC/ASIC family of ion channels [59,60]. This was not obvious from amino acid sequence alignments, and was further thought to be unlikely on the basis of spurious assignments of receptor stoichiometry in the case of degenerins/ENaC/ASIC (see [61]). Thus, discordant avenues of physiological investigation that were quite divergent in 1994 are now moving together. Not only has the ‘P2X prodigal child’ returned, but a family who hardly knew each other has been reunited. It is fully to be expected that they will spend the next period getting to know each other, and thus elucidating their common features of molecular operation.

## Concluding remarks

Current interpretation of the functional properties of P2X receptors in terms of atomic structure will presage a much more detailed understanding of how ligands bind to these receptors. Determining the structure of the open channel, the channel bound with ATP, and even the dilated channel, will be important. Structure-based drug design and computational methods can now be used to address the paucity of small molecule pharmacological tools that has handicapped progress towards a fuller understanding of function. The differences in structure among the seven mammalian P2X receptor subunits can be used to develop subunit specific antagonists. We must hope that some of these tools can be developed into new therapeutics, particularly in neuropathic pain and inflammation [62,63].

## Figures and Tables

**Table 1:** Summary of the major physiological functions of P2X receptors

Receptors	Functions
P2X1	Contraction of the vas deferens and male fertility [64], renal microvascular autoregulation [65], thrombosis [66], and neutrophil chemotaxis [67]
P2X2, P2X3 or P2X2/3	Inflammatory and neuropathic pain [68–70], urinary bladder reflex [68], enteric neurotransmission and peristalsis [71,72], chemoreception [73,74], and taste transduction [75]
P2X4	Neuropathic pain [76], long-term potentiation in the hippocampus [77], and vascular tone and remodelling [78]
P2X7	Cytokine release [79], bone remodelling [80], inflammatory and neuropathic pain [81,82], collagen deposition and renal fibrosis [83], and glia–neuron interactions [84,85]

**Table 2:** ATP-binding residues. For eight residues positioned at the ATP-binding pocket, substitutions reduce the effectiveness of ATP to open the P2X receptor channel expressed in heterologous cells. Δ indicates the log_10_ of the change in concentration of ATP causing half-maximal current (EC_50_), >4 indicates <10% of wild-type current even at 3–10 mM ATP, 300 μM BzATP or αβmeATP. ^a^P2X3 receptors tested with αβmeATP.

P2X1	Δ		P2X2	Δ	P2X3^a^	Δ	P2X4	Δ	P2X7	Δ	References
K68A	3.2		K69A	>4	K63A	>4	K67A	>4	K64A	>4	29,30,86,87,88,89
K68R	>4		K69C	>4			K67C	>4			
			K69R	2.5			K67R	>4			
K70A	0.7		K71A	2.7	K65A	1.1	K69C	>2.4			30,86,87,88
K70R	1.3		K71C	3.0							
			K71R	0.6							
F185A	1.0		F183C	0.5			F185A	1.0			87,89,90,91
F185C	0.3						F185C	1.3			
							F185W	0.3			
T186A	0.8		T184A	2.1			T186C	1.7			30,87,91,92
T186C	0.9		T184C	1.0							
N290A	1.8		N288A	2.0			N293C	>2.4			30,87,92,93
N290C	1.9		N288C	>2.4							
F291A	2.2		F289C	0.7			F294A	0.9			87,89,90,93
F291C	1.7						F294W	0.6			
R292A	2.0		R290A	2.4	R281A	>1.8	R295A	>3			30,86,87,88,89,93
R292C	1.3		R290C	>2.4			R295K	>3			
R292K	2.1		R290K	0.9							
K309A	3.2		K308A	>4	K299A	>4	K313A	>3			30,86,88,93,94,95,96
K309C	2.3		K308C	>4			K313R	>3			
K309R	1.4		K308R	1.5							
